# Evaluating Closures of Fresh Fruit and Vegetable Vendors During the COVID-19 Pandemic: Methodology and Preliminary Results Using Omnidirectional Street View Imagery

**DOI:** 10.2196/23870

**Published:** 2021-02-18

**Authors:** Shahmir H Ali, Valerie M Imbruce, Rienna G Russo, Samuel Kaplan, Kaye Stevenson, Tamar Adjoian Mezzacca, Victoria Foster, Ashley Radee, Stella Chong, Felice Tsui, Julie Kranick, Stella S Yi

**Affiliations:** 1 Department of Social and Behavioral Sciences School of Global Public Health New York University New York, NY United States; 2 Environmental Studies Program Binghamton University, State University of New York New York, NY United States; 3 Department of Population Health NYU Grossman School of Medicine New York, NY United States; 4 Chatham High School Chatham, NJ United States; 5 Mailman School of Public Health Columbia University New York, NY United States

**Keywords:** built environment, Google Street View, food retail environment, COVID-19, geographic surveillance, food, longitudinal, supply chain, economy, demand, service, vendor, surveillance

## Abstract

**Background:**

The COVID-19 pandemic has significantly disrupted the food retail environment. However, its impact on fresh fruit and vegetable vendors remains unclear; these are often smaller, more community centered, and may lack the financial infrastructure to withstand supply and demand changes induced by such crises.

**Objective:**

This study documents the methodology used to assess fresh fruit and vegetable vendor closures in New York City (NYC) following the start of the COVID-19 pandemic by using Google Street View, the new Apple Look Around database, and in-person checks.

**Methods:**

In total, 6 NYC neighborhoods (in Manhattan and Brooklyn) were selected for analysis; these included two socioeconomically advantaged neighborhoods (Upper East Side, Park Slope), two socioeconomically disadvantaged neighborhoods (East Harlem, Brownsville), and two Chinese ethnic neighborhoods (Chinatown, Sunset Park). For each neighborhood, Google Street View was used to virtually walk down each street and identify vendors (stores, storefronts, street vendors, or wholesalers) that were open and active in 2019 (ie, both produce and vendor personnel were present at a location). Past vendor surveillance (when available) was used to guide these virtual walks. Each identified vendor was geotagged as a Google Maps pinpoint that research assistants then physically visited. Using the “notes” feature of Google Maps as a data collection tool, notes were made on which of three categories best described each vendor: (1) open, (2) open with a more limited setup (eg, certain sections of the vendor unit that were open and active in 2019 were missing or closed during in-person checks), or (3) closed/absent.

**Results:**

Of the 135 open vendors identified in 2019 imagery data, 35% (n=47) were absent/closed and 10% (n=13) were open with more limited setups following the beginning of the COVID-19 pandemic. When comparing boroughs, 35% (28/80) of vendors in Manhattan were absent/closed, as were 35% (19/55) of vendors in Brooklyn. Although Google Street View was able to provide 2019 street view imagery data for most neighborhoods, Apple Look Around was required for 2019 imagery data for some areas of Park Slope. Past surveillance data helped to identify 3 additional established vendors in Chinatown that had been missed in street view imagery. The Google Maps “notes” feature was used by multiple research assistants simultaneously to rapidly collect observational data on mobile devices.

**Conclusions:**

The methodology employed enabled the identification of closures in the fresh fruit and vegetable retail environment and can be used to assess closures in other contexts. The use of past baseline surveillance data to aid vendor identification was valuable for identifying vendors that may have been absent or visually obstructed in the street view imagery data. Data collection using Google Maps likewise has the potential to enhance the efficiency of fieldwork in future studies.

## Introduction

The COVID-19 pandemic has evolved into one of the most significant and socially disruptive health crises in recent history, with growing concern for how food systems at both the global and local levels are being affected by the dramatic economic and social impacts of the pandemic [1]. Given the significance of the retail food environment in fostering and maintaining healthy diets [2], disruptions to certain components of this environment, such as access to fresh fruits and vegetables, have the potential to detrimentally impact population health, which has already been identified as an area of concern [3]. However, early observational evidence from Google Search frequency data suggests greater search-based interest in fresh foods during the COVID-19 pandemic [4], which supports shopping behavior data showing a significant increase in fresh fruit and vegetable sales during the early months of the pandemic (although demand is stabilizing) [5]. Moreover, social distancing measures are significantly impacting direct sources of fresh produce, such as farmers’ markets [3].

Although national and international supermarket chains, warehouse clubs, and supercenters dominate grocery retail [6], fruits and vegetables are sold in a variety of other food retail environments as well. Fresh fruit and vegetable vendors are often smaller and more community-oriented than other restaurant or retail food outlets, and can include chain or independent grocery stores, greengrocers, storefront stands (or simply “storefronts,” which are areas in front of stores used to sell fresh fruits and vegetables), street carts, and even makeshift platforms focused on the sale of fresh fruits and vegetables [7]. Although fresh produce—as opposed to processed produce (ie, canned, dried, or frozen produce that can often be found in other larger food retailers)—may not have a substantially higher nutritional value [8] (and fresh produce may also be purchased at these larger food retailers [9]), smaller community fresh fruit and vegetable vendors who may conduct business on the sides of major streets or on storefronts have played an integral role in the food environment in large urban centers such as New York City (NYC) [10], particularly in ethnic enclaves where they represent a significant source of fruits and vegetables [7].

Many fresh fruit and vegetable vendors (particularly street carts selling fresh fruits and vegetables) in cities across the United States, including NYC, have been forced to close since the COVID-19 pandemic began due to the dual concerns of plummeting demand and fear of contracting COVID-19 [11,12]. Within cities, the importance and presence of fresh fruit and vegetable vendors varies by neighborhood. In Manhattan’s Chinatown, fresh fruit and vegetable vendors are known for their low prices and play a significant role in the food retail environment as a critical food source for socially and economically vulnerable populations (eg, the elderly [13]), which have been a growing proportion of Manhattan’s Chinatown demographic makeup. Furthermore, these fresh fruit and vegetable vendors are magnets for tourists and interborough shoppers from diverse cultural backgrounds—not only Asian backgrounds—who are looking for items that cannot be found elsewhere in the city, or for the same low costs [7].

Unlike larger, well-established grocery store vendors, these fresh fruit and vegetable vendors (which are relatively smaller and more community-centered) may not have the financial infrastructure to withstand the changes in supply and demand induced by the COVID-19 pandemic [14]; thus, the risk of closure or changes in services may be more significant for these vendors. Likewise, many fresh fruit and vegetable vendors in NYC are immigrants [7,15], often with low English proficiency [7], and thus may not have the same social and economic capital or legal protections that enable the retail resiliency of other vendors. Therefore, understanding how the pandemic has impacted fresh fruit and vegetable vendors can provide vital insights into changes in fresh fruit and vegetable food environments in large urban centers, such as NYC, where these community-based vendors are a significant part of the fresh produce environment.

Evaluating the impact of the COVID-19 pandemic on services provided by fresh fruit and vegetable vendors fundamentally requires surveillance data both before and after the onset of the pandemic. Given that updated surveillance data of the diverse types of community fresh fruit and vegetable vendors may not be available (particularly for the more informal, street-based vendors), Google Street View is a promising platform for data collection in this context. Google Street View has been employed in a variety of health research contexts [16], including assessing local food environments [17,18], and is a less resource-intensive, easily accessible source of visual data in comparison to other forms of visual data collection, such as those relying upon in-person fieldwork [16]. Google Street View data is usually collected from cars sent out by Google, which are equipped with cameras with the ability to capture 360-degree views in a particular location. Image data is then geotagged and uploaded on Google platforms (such as Google Maps) accessible for public use [19]. Importantly, these Google Street View images are routinely updated. Large urban centers in high-income countries (eg, NYC) often have more recent Google Street View imagery data in comparison to other locations [16], which is likely because a location's population density contributes to the frequency of street view imagery updates conducted by Google [19]. For example, in June 2020, NYC Google Street View imagery data from June and October 2019 could be accessed.

In the past, Google Street View has been used for cross-sectional or validation-based study designs, and thus the use of the platform for longitudinal health research in neighborhood settings is an area in need of more exploration [16]. Likewise, while longitudinal analysis of Google Street View data has been effective in retrospectively analyzing changes in food retail environments, the use of the platform to analyze more recent changes by triangulating information from the platform with other means of surveillance remains underexplored [20].

Therefore, the aim of this study is to document the methodology developed to assess changes in the food retail environment (notably, closures of fresh fruit and vegetable vendors) before and during the COVID-19 pandemic in NYC using triangulated data from Google Street View, past surveillance (when available), and Google Maps–based, socially distanced in-person assessments. Specifically, we document the following: (1) the specific procedures used to conduct fresh fruit and vegetable surveillance both before and during the pandemic such that they may be replicated by others in other locations, and (2) the strengths and limitations of this methodology, as well as the challenges faced. Importantly, as opposed to analyzing general net changes in the fresh fruit and vegetable food retail environment (which has been explored for other food retail environments using street view data [20]), a specific focus of this methodology was to analyze closures and other visually observable service impacts on pre-existing vendors directly prior to and during disruptive crises such as the COVID-19 pandemic.

## Methods

### Baseline Fresh Fruit and Vegetable Vendor Assessment: Past Surveillance Data

NYC is divided into five boroughs, which each contain many neighborhoods. The boroughs of Manhattan and Brooklyn were analyzed in this study. Neighborhoods were defined using NYC Neighborhood Tabulation Areas, and further details on their identification have been described elsewhere (RG Russo et al, unpublished data, July 2020; SS Yi et al, unpublished data, July 2020); in short, data on the socioeconomic and health disparities of Manhattan and Brooklyn neighborhoods were used to select one socioeconomically advantaged neighborhood (Upper East Side, Park Slope), one socioeconomically disadvantaged neighborhood (East Harlem, Brownsville), and one Chinese ethnic neighborhood (Chinatown, Sunset Park) in each borough. An ethnic Chinese neighborhood was selected for analysis in each borough given observational evidence identifying the strong role fresh fruit and vegetable vendors play in the fruit and vegetable retail environments within Chinese ethnic neighborhoods in NYC [7]. Past surveillance data on the locations and types of fresh fruit and vegetable vendors in the select 3 neighborhoods in Manhattan (Chinatown, Upper East Side, and East Harlem) and 3 neighborhoods in Brooklyn (Sunset Park, Park Slope, and Brownsville) were first identified to establish the baseline fresh fruit and vegetable vendor landscape from which prepandemic and pandemic assessments were to be conducted.

Identifying past surveillance data to guide the prepandemic fresh fruit and vegetable vendor identification was important for two reasons. First, one of the key limitations of Google Street View data extraction is that visual obstructions in the Google Street View image may conceal fresh fruit and vegetable vendors (eg, in the NYC Chinatown extraction, trucks parked in the street could make sidewalks and some smaller vendors, such as makeshift platforms, difficult to view or notice). Therefore, the non–Google Street View surveillance data allows one to account for these visually obstructed and inconspicuous vendors. Second, some fresh fruit and vegetable vendors in cities such as NYC operate in a temporary capacity, with some operating in makeshift physical locations in areas such as parking lots. Therefore, integrating past cross-sectional non–Google Street View surveillance data with visual data from Google Street View aids in identifying long-term vendors and allows for a more comprehensive, precise evaluation of how the COVID-19 pandemic has impacted established fresh fruit and vegetable vendors that operate in a more sustained capacity within the community.

Imbruce [7] collected in-depth fresh fruit and vegetable surveillance data in Chinatown, which was used as the baseline fresh fruit and vegetable vendor assessment data for this neighborhood, given the diversity of fresh fruit and vegetable vendors and specificity of geographic information included in the data set. Equivalent information was not available for the other five neighborhoods. However, Fuchs et al [15] did collect geographic data on a small percentage of Green Carts (mobile street vendors specially permitted to sell exclusively fresh fruits and vegetables) in East Harlem and Brooklyn. Therefore, this information was used as a guide to identify the potential locations of fresh fruit and vegetable vendors in the prepandemic assessment of these neighborhoods, using Google Street View to concretely identify fresh fruit and vegetable vendor locations. Importantly, given this past surveillance data was quite dated (ranging from 2003 to 2013) and that fresh fruit and vegetable vendors are likely to have changed between then and 2019, this supplementary surveillance data was largely used to identify any additional potential vendors or locations of past clusters of vendors during in-person checks; additional vendors identified during in-person checks may have been missing or visually obstructed in the 2019 street view imagery data. For the Upper East Side, Park Slope, and Sunset Park, for which there were no comprehensive or reliable baseline fresh fruit and vegetable vendor assessment data sources, Google Street View was solely relied upon to identify prepandemic vendors (Table 1).

**Table 1:** Data sources used to assess COVID closures of fresh fruit and vegetable vendors.

Neighborhood	Baseline data	Prepandemic data	Data during the pandemic
Chinatown	2003-2005 (Imbruce, 2015 [7])	June-October 2019 (Google Street View)	June-July 2020 (in-person)
Upper East Side	None	June-October 2019 (Google Street View)	June-July 2020 (in-person)
East Harlem	2013 (Fuchs et al, 2014 [15])^a^	June-October 2019 (Google Street View)	June-July 2020 (in-person)
Sunset Park	None	June-October 2019 (Google Street View)	June-July 2020 (in-person)
Park Slope	None	June-October 2019 (Google Street View)^b^	June-July 2020 (in-person)
Brownsville	2013 (Fuchs et al, 2014 [15])^a^	June-October 2019 (Google Street View)	June-July 2020 (in-person)

^a^Since geographic surveillance data only captured 45/121 of the Green Carts given permits in Manhattan and 19/132 of those in Brooklyn, and specific location data was not available, this data source was only used to provide a general understanding of potential prior locations.

^b^Some streets did not have Google Street View data from 2019, and these areas were supplemented with street-view surveillance from Apple Look Around.

To allow for disaggregated analyses, fresh fruit and vegetable vendors identified in either the baseline or prepandemic assessments were categorized into four types (store/supermarket, storefront, street vendor/other, and wholesale) based on criteria identified by Imbruce [7] (Table 2). Although some of the terminology for these categorizations has been employed in different ways in past research [21], for the purposes of this study, the definitions provided by Imbruce, which were based on in-depth fieldwork in NYC’s Chinatown, were used to define different fresh fruit and vegetable vendors.

**Table 2:** Types of fresh fruit and vegetable vendors analyzed, adapted from a study by Imbruce [7].

Type	Description
Store/supermarket	Both the sidewalk in front of the store and the inside of the store are used primarily for selling produce (operated by same owner).
Storefront	Only the sidewalk in front of a store is used to sell produce.
Street vendor/other	Produce sold in areas of high foot traffic in spaces near streets, sometimes by itinerant vendors. Other types of fresh fruit and vegetable vendors (eg, outdoor markets, makeshift stores) were also included.
Wholesale	Produce is bought from farms or other produce brokers in high volumes and is sold to retail vendors, restaurants, or individuals by the box.

### Prepandemic Fresh Fruit and Vegetable Vendor Assessment: Google Street View Extraction

A spreadsheet was created using the data points identified from the baseline fresh fruit and vegetable assessment. For Chinatown, each baseline data point was given a unique ID number from this prior surveillance data [7], and any other information provided by the survey (eg, street location, category of fresh fruit and vegetable vendor) was included. Given that many fresh fruit and vegetable vendors (notably street carts) may not have visibly identifying features, the identification of these unique fresh fruit and vegetable data points was informed by (1) the specific street address where a particular vendor was located, (2) the type of vendor (eg, if a street cart fresh fruit and vegetable vendor was stationed in front of a store/storefront fresh fruit and vegetable vendor at the same street address, these would each get unique ID numbers). Google Street View was then employed to ascertain whether the fresh fruit and vegetable vendor corresponding with each unique ID was visible (labeled as “found”) from a 2019 Google Street View image at the approximate location identified from the prior surveillance data [7]. For all other neighborhoods, since reliable past surveillance data was not available to guide the Google Street View assessment, research assistants virtually walked down each street of the neighborhoods using Google Street View and catalogued all fresh fruit and vegetable vendors they identified in a spreadsheet along with the approximate street location, date of Google Street View image, and any notes relevant to the vendor’s services or its location.

**Figure 1:**
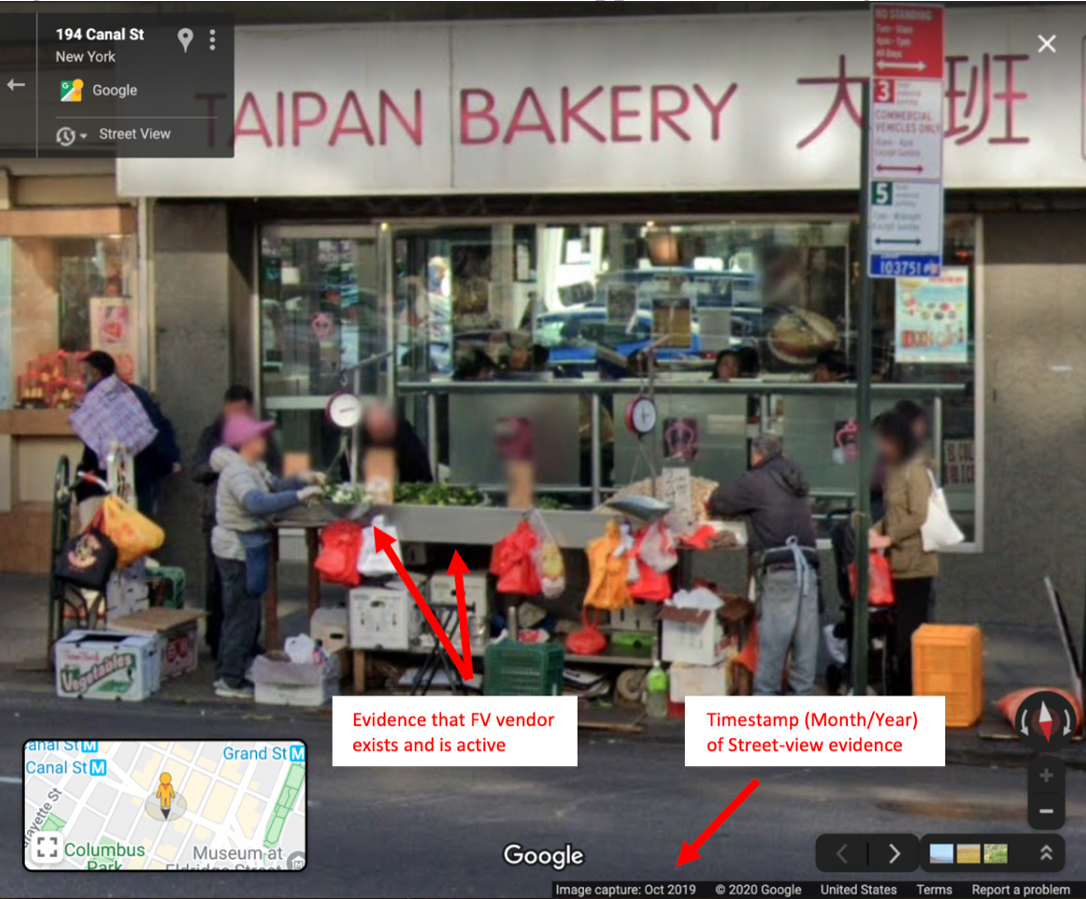
Data extracted from Google Street View to support fruit and vegetable vendor presence. FV: fruit and vegetable.

From the street view image at the location of the baseline site, a fresh fruit and vegetable vendor data point was labeled as “found” if the following was true: (1) the physical location of a fresh fruit and vegetable vendor was able to be clearly identified, based on the description or category of the vendor provided by the prior survey (eg, cart, storefront, wholesaler), and (2) there was visual evidence to support the vendor being active, such as the presence of fruits and vegetables and/or individuals actively engaging in consumer activity (eg, exchanging cash or multiple people holding grocery bags in the vicinity of the location). Fresh fruit and vegetable vendors that did not qualify as being “active” included those with signage indicating ongoing construction or renovation, or an indication that the vendor had not yet formally opened for business (ie, “opening soon”). Vendors with signage that indicated changed services (eg, delivery or takeout only) were classified as “active.” Vendors exclusively selling items other than fruit and vegetables (eg, clothes or toys) were not included. Any fresh fruit and vegetable vendor data point that did not satisfy these conditions was labeled as “not-found”; furthermore, if there was visual evidence to suggest that there was a temporarily or permanently closed fresh fruit and vegetable vendor matching the description from the prior survey at the location of the data point, then these observations were noted in a separate “notes” column in the extraction data sheet. Likewise, any other anomalies regarding the visual Google Street View evidence from the “found” or “not found” fresh fruit and vegetable vendors were noted in this “notes” column. The time stamp of the Google Street View image (month and year) was also catalogued. A visual depiction of the information gleaned from the Google Street View scan is displayed in Figure 1.

Although Google Street View image data across all neighborhoods were largely time-stamped with a 2019 date, some streets in Park Slope were observed to only have Google Street View imagery data from dates prior to 2019. To address this, research assistants collected street imagery data from Apple Look Around, a new geographic imagery system implemented by Apple Inc in 2019-2020, which is analogous to Google Street View [22]. Similar to Google Street View, Apple Look Around relies upon ground surveys conducted by commissioned vehicles to collect geographic imagery data [23]. Select areas of Park Slope without 2019 time-stamped Google Street View data were supplemented with 2019 time-stamped Apple Look Around imagery to complete the data collection for this neighborhood.

### Fresh Fruit and Vegetable Vendor Assessment After the Start of the COVID-19 Pandemic: Google Maps–Based In-Person Checks

Following the 2019 Google Street View data extraction, an in-person check was conducted for each fresh fruit and vegetable vendor between June and July of 2020. All fresh fruit and vegetable vendors, including those that were either “found” or “not found,” received in-person checks to ensure vendors that may not have been found due to the aforementioned limitations of Google Street View imagery were not excluded. A protocol for rapid in-person assessments was designed to specifically incorporate principles of social distancing and minimize outside exposure for research staff. These principles were achieved through two key components of the protocol. First, to minimize the time needed to find the locations of each fresh fruit and vegetable vendor and enhance the speed of data collection, several functions in Google Maps were used. Specifically, the “Your Places” feature was used to create lists containing the pinpoint locations of each fresh fruit and vegetable vendor in a shared Google Maps account. The “notes” feature of each pinpoint was then used to record the unique vendor ID, and other information from the prepandemic assessment stage relevant to the in-person checks (such as the name and category of vendor, as identified from Google Street View or baseline surveillance). From the spatial data of the fresh fruit and vegetable vendor pinpoints, an in-person check route was designed to minimize the amount of walking or driving required. During the in-person checks, research staff accessed Google Maps on their mobile phones using the shared account with the fresh fruit and vegetable vendor lists and pinpoints. Second, measures were taken to also enhance social distancing for research stuff, which (along with the standard government-mandated use of face masks at the time of the visit) included conducting in-person checks by car when possible (largely to identify street cart or storefront vendors that could be identified from a car). Finally, data collection was conducted largely during the afternoon, on different days of the week, and not during periods of inclement weather to minimize the potential of conducting data collection during routine or temporary fresh fruit and vegetable vendor closures.

**Figure 2:**
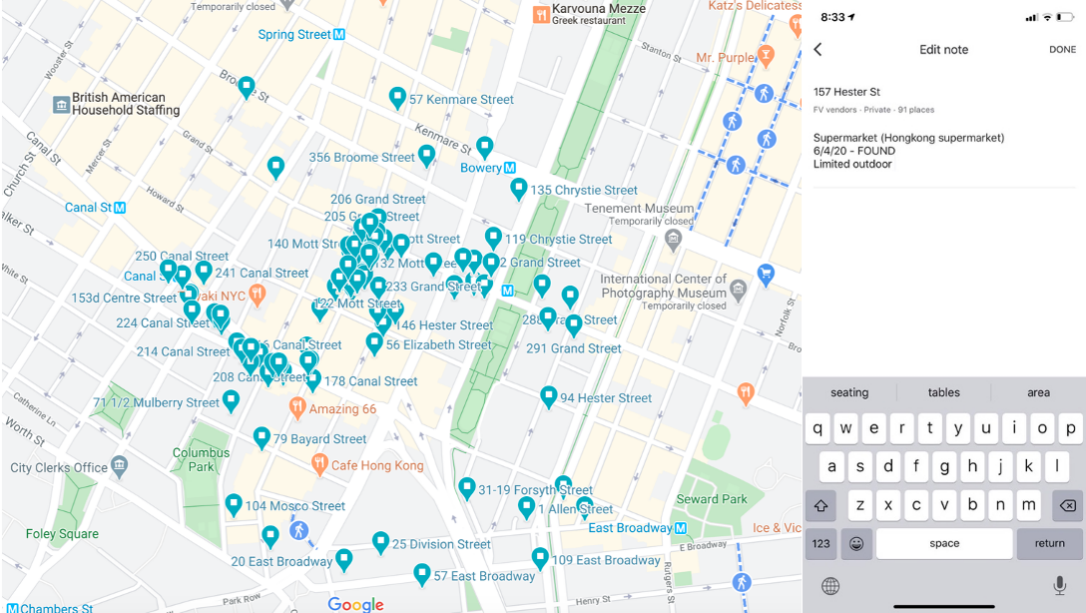
Example of Google Maps pins and the "notes" feature used for in-person checks (Chinatown).

When a pinpoint was reached, research staff used the “notes” feature, which contained the prepandemic information, to catalog the pandemic assessment data, including the following: (1) whether the vendor was found and, if so, whether the vendor was observed to be open, open with a more limited setup, or closed, (2) the date the in-person check was conducted, and (3) any notes about the vendor or its services. Given that the physical location of vendors may have slightly changed (notably street cart vendors), to help in the identification of a particular vendor, research assistants examined all street addresses in close proximity to the noted location (eg, examining a few street numbers to the left and right of the location), as well as information from the prepandemic street view imagery indicating the types of produce being sold at the location. “Open with more limited setups” was noted when vendors appeared to have slightly changed or offered fewer services than what was observed during the Google Street View prepandemic extraction (eg, fresh fruit and vegetable stores closing the outdoor portions of their services, or certain sections of street carts closed or selling fewer quantities or varieties of produce). Examples of notes made during in-person assessments included if a vendor was likely replaced by a new store, or if the vendor was sharing services or affiliating with another vendor. Figure 2 displays an example of the Google Map pinpoints and “notes” feature interface used for the in-person data collection.

Importantly, during data collection, research assistants may have also encountered fresh fruit and vegetable vendors that did not have Google Maps pinpoints based on the baseline and street view imagery data. Given the focus of this methodology was to evaluate closures and other visually observable service impacts on fresh fruit and vegetable vendors, such vendors lacked the prepandemic 2019 street view or baseline surveillance data to be eligible to be included in the longitudinal assessment of fresh fruit and vegetable vendor closures; therefore, systematic data collection was not conducted for these vendors. Nevertheless, throughout data collection, research assistants reported these additional vendors to the study team.

## Results

**Figure 3:**
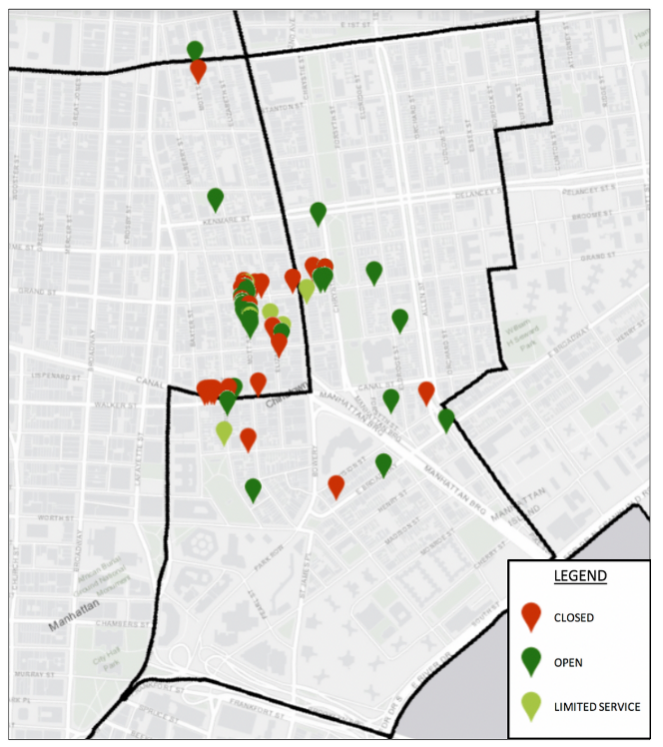
Example of consolidated visual output using data collected from prepandemic and pandemic assessments (Chinatown).

The initial Google Street View extraction (partially informed by baseline data in the case of Chinatown) identified 80 vendors in the three Manhattan neighborhoods: 56 in Chinatown, 12 on the Upper East Side, and 12 in East Harlem. A total of 55 vendors were also identified in the three Brooklyn neighborhoods: 48 in Sunset Park, 4 in Park Slope, and 3 in Brownsville. Although only 53 vendors were identified using Google Street View in Chinatown, during in-person checks, 3 additional vendors were identified that had been noted in the baseline surveillance data but were not found using Google Street View, increasing the total vendor sample of Chinatown to 56. A summary of the preliminary extraction data and COVID-19 pandemic closure data is presented in Table 3. An example of the data visualization methods used to highlight pandemic-related closures is displayed in Figure 3. Overall, 60 of the 135 vendors (44%) identified in all 6 neighborhoods were either absent/closed or had more limited setups following the beginning of the COVID-19 pandemic.

**Table 3:** Preliminary findings on closures of fresh fruit and vegetable vendors in select New York City neighborhoods during the COVID-19 pandemic using Google Street View analysis.

Neighborhood		No change, n (%)	Closure during the pandemic, n (%)	More limited setups during the pandemic, n (%)
**Manhattan**				
	Chinatown (n=56)^a^	27 (48.2)	24 (42.9)	5 (8.9)
	Upper East Side (n=12)	5 (41.7)	0 (0.0)	7 (58.3)
	East Harlem (n=12)	7 (58.3)	4 (33.3)	1 (8.3)
**Brooklyn**				
	Sunset Park (n=48)	30 (62.5)	18 (37.5)	0 (0.0)
	Park Slope (n=4)	3 (75.0)	1 (33.3)	0 (0.0)
	Brownsville (n=3)	3 (100.0)	0 (0.0)	0 (0.0)

^a^Includes vendors found in the 2019 Google Street View check (n=53) and additional vendors found during in-person checks (n=3), which were also identified in other baseline surveillance.

## Discussion

The integrated Google Street View–centered longitudinal assessment of fresh fruit and vegetable vendors was able to identify significant closures among these vendors during the COVID-19 pandemic by comparing evidence from shortly before the pandemic in 2019 and during the pandemic. Although the identified closures cannot all be directly attributed to the COVID-19 pandemic itself using this preliminary surveillance evidence, this approach helped to highlight the extent of pandemic-related closures within a facet of the food retail environment with limited formal, consistent surveillance, which nonetheless plays an integral role in underserved ethnic minority communities, such as those in Manhattan’s Chinatown [7]. Likewise, Google Street View data could be transferred efficiently into Google Maps as pinpoints to facilitate time-efficient and resource-efficient in-person checks. To the best of our knowledge, the use of the “notes” feature of Google Maps has not been explicitly employed as a data collection tool in past health research. Google Maps was able to provide real-time information to multiple members of the study team on which sites had been catalogued; this is evidence that the platform can significantly enhance the efficiency of fieldwork for future studies, particularly in resource- and time-scarce contexts such as the COVID-19 pandemic.

Importantly, use of past baseline surveillance data (when available) to assist in fresh fruit and vegetable vendor identification during the prepandemic assessment was found to be valuable; the identification of 3 vendors in Chinatown that were not found using Google Street View but were found in both the baseline assessment and in-person visits supports preliminary concerns that visually obstructed vendors may be missed in Google Street View imagery. Further use of Google Street View should also consider supplementing assessments with other data sources, particularly when assessing objects that may be susceptible to being missed or obscured in Google Street View imagery [16]. These findings directly support evidence from prior Google Street View research, which similarly identified image quality and visual obstructions as areas of concern [16]. However, unlike many past studies, the date of capture for the analyzed Google Street View imagery was consistently recent (almost always sometime in 2019) [16]; this was likely due to NYC—a large, populated area likely to be frequented by Google-commissioned cars for Google Street View surveillance—being the study setting of this project. Given that large, urban environments have been particularly affected by the COVID-19 pandemic [24], Google Street View may be particularly useful for conducting prepandemic versus pandemic (or postpandemic) assessments in these environments.

Likewise, given the significant disparities in food purchasing behavior—including the quantity and quality of food as well as purchasing frequency and sources of food access—across various minority populations in the United States, the importance of this methodology also lies in its ability to survey aspects of the food retail environment that may be critical for underserved minorities, such as Asian Americans [7]. Although conducting prepandemic/pandemic closure assessments for storefront food vendors (eg, restaurants or grocery stores) can be done through the use of a variety of sources of information, such as the internet (eg, Yelp, Google, social media) or publicly available phone numbers (RG Russo et al, unpublished data, July 2020), fresh fruit and vegetable vendors are relatively disconnected from public information databases as they cater to localized populations and may operate less formally than other food retail outlets. Given these fresh fruit and vegetable vendors both serve and are often managed by vulnerable minority populations [7,15], understanding the significance of this methodology with respect to health equity concerns in food retail environment surveillance is paramount.

Nonetheless, there were a number of limitations faced throughout the study. First, it is important to acknowledge that some vendors may have been closed for the day at the time of Google Street View image capture or in-person checks, which may have led to an overestimation of pandemic-related closures. These potential routine or temporary closures have particularly impacted the assessment of street vendors (as opposed to stores or storefronts, which may have more established operating hours or more staff to assist in maintaining consistent operations). These concerns were mitigated in two ways: (1) baseline data helped to corroborate any vendors that might have been missed in Google Street View extraction, and (2) in-person checks were conducted at times of the day when most food retail vendors (including fresh fruit and vegetable vendors) are open (ie, afternoon, early afternoon).

Moreover, while steps were taken to assist in-person checks of fresh fruit and vegetable vendors that may have a slightly changed geographic location, some vendors may have moved to an entirely different street or completely changed their services between 2019 and 2020. Likewise, while the sample of vendors included in the prepandemic assessment was maximized by using recent (largely June and October 2019) imagery data along with supplemental past surveillance data to identify established fresh fruit and vegetable vendors that may have been obstructed or missed in street view imagery but were present in in-person checks, some vendors that been missed by these data sources or that had opened later in 2019 but still prior to the impact of the COVID-19 pandemic in the United States were unable to be examined. However, while systematic data collection of additional vendors identified during in-person checks was not conducted, research assistants did not report more than a few additional fresh fruit and vegetable vendors in each neighborhood outside of the prepandemic sample, in part due to the aforementioned efforts as well as the short time frame between the prepandemic data assessments and those obtained during the pandemic. Nonetheless, while examining net changes in the food retail environment was not a focus of this particular methodology, the incorporation of systematic analysis of new vendor openings after disruptive crises such as the COVID-19 pandemic is another area worthy of exploration (including across different types of food retail outlets).

Finally, it is likely that some fresh fruit and vegetable vendors may have closed for some time early on in the pandemic but may have recently reopened. Alternatively, vendors may have opened shortly after the particular day in-person checks were conducted, but still within the June-July 2020 endpoint time frame. This is a limitation of the approach; to provide the most accurate, cross-sectional COVID-19 pandemic surveillance data, data collection must occur within a short period of time. In this case, due to the novelty of the methodology, many of its components were being developed and tested by the study authors throughout each stage, thus limiting the speed of the in-person checks. Likewise, due to the COVID-19 pandemic, caution also had to be taken in the timing of the in-person checks to minimize outdoor exposure for research staff. However, we intend on doing targeted follow-up assessments in subsequent months.

Indeed, the methodology described in this study has significant implications for research aimed at longitudinally assessing recent closures in the food retail environment (particularly among fresh fruit and vegetable vendors or other retailers with limited public surveillance data) during time- or resource-sensitive time frames, including disruptive health crises such as the COVID-19 pandemic. For example, the impact of the COVID-19 pandemic on the fresh fruit and vegetable retail environment has been felt by other communities across the United States; fresh fruit and vegetable vendors in Los Angeles have witnessed a dramatic drop in sales [25], which may be catalyzing vendor closures. Moreover, to address the sales and logistical disruptions faced by many fresh fruit and vegetable vendors, new online-based delivery services for these vendors have been explored in limited settings [26]; future research may involve surveillance of different strategies being explored by fresh fruit and vegetable vendors to adapt services and prevent closures. Likewise, it is important to contextualize the potential economic or food access impacts related to fresh fruit and vegetable vendor closures identified in this methodology with the broader health or social impacts of the COVID-19 pandemic; complementing this methodology with other mixed methods approaches to assess the economic, health, and social impacts of the COVID-19 pandemic is warranted. Finally, to the best of our knowledge, this study was the first to use the new Apple Look Around geographic data system for health research. The platform was observed to be efficient and user-friendly in identifying fresh fruit and vegetable vendors in Park Slope in a manner similar to the use of Google Street View; however, since the platform was not used in the other examined neighborhoods, further research is needed to assess its effectiveness for health research both in NYC and in other settings. With these preliminary insights from Park Slope, future in-depth analysis comparing the utility of Apple Look Around with Google Street View and other means of geographic surveillance is warranted, particularly with respect to parameters such as image quality, geographic scope of the data, timeliness of surveillance updates, and other features of the platform that can assist in health research.

## Abbreviations

NYC: New York City
